# Unexplored Questions About Away Rotations – A Student’s Perspective

**DOI:** 10.15694/mep.2019.000019.1

**Published:** 2019-01-23

**Authors:** Jeremy Light, Santiago Gonzalez, Michael Franzetti

**Affiliations:** 1 University of Arkansas for Medical Sciences

**Keywords:** Away rotation, medical school, residency, NRMP Match, competitive specialties

## Abstract

This article was migrated. The article was marked as recommended.

Many fourth-year medical students complete away rotations as elective courses within their specialty of choice. The popularity of away rotations is increasing and has become nearly ubiquitous in certain competitive specialties ( Higgins et al., 2016). Benefits include obtaining letters of recommendation, diversifying clinical exposure, expanding research opportunities, and establishing a connection with a residency program. Some disadvantages include cost, stress, competitive nature among applicants, and separation from personal support networks. We are writing this to bring attention to the pros and cons of away rotations, and to the absence of substantial data available in the literature to help us understand the actual impact that away rotations may have on our residency matching outcomes.

## Introduction

Graduating medical students complete an average of 1.8 away rotations in their fourth year of medical school ( Higgins et al., 2016). Some specialties such as emergency medicine make it a mandatory task ( King & Kass, 2017). In other specialties, and specifically in highly competitive ones, there is an *unspoken rule* that makes away rotations an additional requirement to successfully match. Regardless of whether away rotations are mandatory, as medical students, we feel pressured to attend a minimum of 2 rotations to demonstrate our commitment to the field we are pursuing.

## Advantages

There are significant advantages to participating in away rotations - the most important being the ability to establish a connection with a program. Factors such as “prior personal knowledge of the applicant” and “away rotation within the department” has been ranked highly by program directors as essential factors in selecting applicants to interview and rank (National Resident Matching Program, 2018). Obtaining a letter of recommendation from a faculty member outside of their home institution may incentivize students who choose to do away rotations. Students interested in a specialty not available at their institution may also benefit from an away rotation, as it is the only means for obtaining letters of recommendation within their specialty of choice. Further opportunities to become involved in research activities may also be an additional benefit. Students with exceptional clinical and interpersonal skills may increase their chances of interviewing by going on away rotations, which may be of particular significance for applicants with lower grades and USMLE scores. Applications can also be screened based on the applicant’s geographical location ( Nagarkar & Janis, 2018). Historically, applicants tend to be interested in programs that are not geographically distant; therefore, an away rotation at a program in a distant geographical area can increase the chances of getting an interview ( Nagarkar & Janis, 2018). Attending a clerkship at another institution allows students to diversify their exposure to clinical practices and teaching styles. It also aids in determining if the applicant’s personality and career goals are a good fit for the program, which is a crucial component when creating a rank list.

## Disadvantages

Although there are many advantages of away rotations, they do come at a price. The cost of transportation, housing, applications, and institutional fees add up to large sums of money. A medical student spends an average of $2,000 on each away rotation; however, it is not unusual for a student to spend as much as $5,000-$10,000 on a rotation ( Winterton, Ahn, & Bernstein, 2016). These costs may create disadvantages for those students who cannot afford the financial burden of away rotations. In addition to financial hardships, students with introverted personalities who may not be accustomed to being “on” the entire time, may be negatively affected by away rotations. Being away from one’s support network, and the competitive nature of fellow applicants may also contribute to the mental and emotional toll experienced by applicants.

## Perspective

It is clear that away rotations are an asset to applicants, even when taking into account the costs and potential disadvantages. Although some data is available to explain the impact that away rotations have on residency matching outcomes, many questions remain unanswered. For example, a study investigating the effects of away rotations on matching outcomes found that 71% of students who did away rotations matched at one of their top three choices ( Higgins et al., 2016). Interestingly, 84% of students who did not do away rotations matched at one of their top three choices ( Higgins et al., 2016). The same study showed that 39% of students matched in an institution at which they performed an away rotation ( Higgins et al., 2016). If participating in away rotations does not increase an applicant’s chances of matching at their preferred programs, then why do so many students choose to participate in them?

As applicants, we would like to bring attention to the need for specialty-specific data to accurately advise students interested in particular fields. The study mentioned above showed no correlation between away rotations and matriculation into their preferred program, but it is unclear if this holds for highly competitive specialties. Due to the lack of comprehensive data on the topic, it may be possible that some students are attending away rotations based on unsupported expectations. A comprehensive understanding of the benefits and disadvantages of away rotations should be a priority because the decision to do an away rotation requires significant time, money, and, for some, higher levels of stress.

Regardless of the lack of information available on the subject, intangible benefits of the face-to-face interactions that away rotations provide would be difficult to replace with other methods. Benefits such as the building of relationships and the feeling of belonging to a program will continue to be valuable to both applicants and residency programs alike.

## Take Home Messages

• There is an increasing number of medical students performing away rotations, particularly in competitive specialties.
• Both advantages and disadvantages exist for students participating in away rotations that should be evaluated on an individual basis.
• There is insufficient data on the impact away rotations have on residency matching outcomes.

## Notes On Contributors

**Jeremy Light** is a third-year medical student at the University of Arkansas for Medical Sciences. ORCID: https://orcid.org/0000-0002-2533-4336

**Santiago Gonzalez** is a third-year medical student at the University of Arkansas for Medical Sciences. ORCID: https://orcid.org/0000-0003-1190-0218

**Michael Franzetti** is a third-year medical student at the University of Arkansas for Medical Sciences.
