# Supplementary material for: Unexplored Questions About Away Rotations – A Student’s Perspective
Source: MedEdPublish (2016). 2019 Jan 23;8:19. [Version 1] doi: 10.15694/mep.2019.000019.1 (PMC10712533; doi:10.15694/mep.2019.000019.1)
Supplement: Peer review history

# Reviewer report: Reviewer response for version 1

**DOI:** 10.21956/mep.19764.r29675

**Published:** 2019-01-25

**Authors:** David Bruce

**Affiliations:** 1 None

This review has been migrated. The reviewer awarded 3 stars out of 5 This opinion piece written by medical students considers the value of away rotations taken in the 4th year and makes a call for more information to be available as to which away rotations will be of most value to students in their applications for specialty residency programmes. The authors give a balanced view of the pros and cons of away rotations and reference previous studies on the subject. This is a well written piece and deals with an important topic that is clearly a concern to students. I feel this paper could be improved in a number of ways. As a reader from outside the USA, a short description of the four years of medical school and the purpose in particular of the third and fourth years would have been helpful background. The authors comment on the paucity of literature, but the studies that they have discussed have up to 30 references including a literature review of the purpose of the fourth year. Perhaps a wider look at the literature would be helpful to readers.The aims of the year (which I am surmising from my reading of the references) are to allow students to explore career interests, broaden their educational experiences and prepare for residency working. This then seems not fully congruent with the students’ main concern that the only way to positively influence their chances of selection into the residency programme of their choice is by undertaking what has been called audition electives. The plot thickens further when the literature does not give a clear correlation between away elective and success in selection to residency programmes. This then gives the student body a real dilemma which could be explored further - perhaps a case study or vignette to illustrate. Cost to the students could likewise be further explored as this does influence who can do what. Finally, having highlighted the problem, I would have liked to have heard the students’ views on how to take this forward. Their piece is well balanced - but an opinion piece perhaps could offer practical suggestions. The students are to be congratulated however for this very readable and balanced article covering an area of concern. I look forward to seeing how this discussion moves forward.

Reviewer Expertise:

NA

No decision status is available

# Reviewer report: Reviewer response for version 1

**DOI:** 10.21956/mep.19764.r29676

**Published:** 2019-01-25

**Authors:** Trevor Gibbs

**Affiliations:** 1 AMEE

This review has been migrated. The reviewer awarded 4 stars out of 5 As my co-reviewer points out, I was also a little confused as to the maening of away-rotations, assuming them to be what others might describe as electives. However, it was good to read a paper from medical students on the subject and how they understand the concept.Prior to the 1993 version of the Tomorrow's Doctors document, electives during the undergraduate medical curriculum were very variable. After being taken over by Student Selected Modules (SSMs) , which eventually were described as Student Selected Components (SSCs) it was perhaps an assumption that Electives took a back seat within curricula. How and where they exist now in the global variation of curricula I feel is poorly understood as well as the benefits they bring to student learning. Hence this paper, which describes the perception of what the learning outcome of these electives are, is a paper worth reading. It could have been greatly enhanced by explaining electives more fully and looking at whether this model was common among other schools, but I do feel it is a paper that curricula developers should read.

Reviewer Expertise:

NA

No decision status is available

# Reviewer report: Reviewer response for version 1

**DOI:** 10.21956/mep.19764.r29677

**Published:** 2019-01-23

**Authors:** P Ravi Shankar

**Affiliations:** 1 American International Medical University

This review has been migrated. The reviewer awarded 4 stars out of 5 I read this manuscript by three third year medical students with great interest. The authors could define the term ‘away rotations’ initially in the manuscript. Are international electives also considered as ‘away rotations’? The authors rightly mention about the scarcity of data with regard to the impact of away rotations. Availability of this data could guide students with regard to the feasibility and impact of doing away rotations. The article will be of interest to a broad range of educators and students.

Reviewer Expertise:

NA

No decision status is available
